# Numerical Analysis of Guided Waves to Improve Damage Detection and Localization in Multilayered CFRP Panel

**DOI:** 10.3390/ma15103466

**Published:** 2022-05-11

**Authors:** Mastan Raja Papanaboina, Elena Jasiuniene, Egidijus Žukauskas, Liudas Mažeika

**Affiliations:** 1 Prof. K. Baršauskas Ultrasound Research Insititute, Kaunas University of Technology, K. Baršausko St. 59, LT-51423 Kaunas, Lithuania; elena.jasiuniene@ktu.lt (E.J.); e.zukauskas@ktu.lt (E.Ž.); liudas.mazeika@ktu.lt (L.M.); 2 Department of Electronics Engineering, Kaunas University of Technology, Studentu St. 50, LT-51368 Kaunas, Lithuania

**Keywords:** guided waves, ultrasonic testing, structural health monitoring, CFRP, CWT, IFFT

## Abstract

Multilayered carbon fiber-reinforced polymers (CFRP) are increasingly used in aircraft components because of their superior mechanical properties. However, composite materials are vulnerable to impact loads, resulting in delamination-type damage which, if unnoticed, could lead to catastrophic structural failure. The objective of this research was to investigate possibilities to improve damage detection and the localization using signal processing methods. Numerical modeling using the semi-analytical finite element (SAFE) method was performed to obtain guided wave dispersion curves and to perform modal analysis. From the modal analysis, $A_{0}$ mode for inspection of the composite with delamination type defects was selected. From the numerical simulation, $A_{0}$ mode interaction with delamination along the longitudinal direction was analyzed and the location of the defect was estimated by measuring the time of flight (ToF) of the signal using Hilbert transform (HT) and continuous wavelet transform (CWT). The CWT has shown better results in estimating the delamination location compared with HT. The depth of delamination was characterized in the frequency domain by comparing the amplitude of the $A_{0}$ mode. Inverse fast Fourier transform (IFFT) is recommended to reconstruct the reflected and transmitted modes for better damage detection and to reduce the complexity of signal interpretation.

## 1. Introduction

Composite materials such as carbon fiber-reinforced polymers (CFRP) and glass fiber-reinforced polymers (GFRP) are widely used in aerospace components, wind turbine blades, and other engineering structures [1,2,3]. The use of multilayered CFRP materials has increased significantly in the aerospace sector due to the high strength-to-weight ratio and because these materials do not corrode. Ultimately, aircraft weight can be reduced, and in return, the reduction of airframe weight allows fuel economy and a reduction of flight operation costs.

However, multilayered CFRP structures are vulnerable to delamination and fiber breakages. Delamination-type defects due to impact damage can occur during assembly and exploitation. Key issues in aircraft wing structures are delamination and disbond-type damage. Such types of defects are difficult to detect during inspection, and this can lead to catastrophic structural failure.

In aerospace applications, structural health monitoring (SHM) could increase the safety, reliability, and quality control of the structure [2]. However, there is no universal SHM technique that can effectively solve all problems. Generally, each SHM technique has its advantages and disadvantages depending on the problem. Among potential SHM techniques such as vibration-based and acoustic emission, ultrasonic-guided wave techniques are widely investigated due to their better damage detection and localization [4]. Different composite materials are increasingly used in aircraft structures [5]. The most common damage to composite materials is impact damage, which causes delamination, disbond, fiber breakage, and matrix cracking [5]. Impact damage using guided waves was investigated by Diamanti et al. using the A_0_ mode [1,5], by Capriotti et al. using noncontact air-coupled guided wave excitation [6], and by Memmolo et al. using permanently installed sensors and the multiparameter approach [6,7]. The multilevel approach for the investigation of impact damage detection and localization was proposed by Khodaei [8]. The interaction of guided waves with delamination-type defects was studied by several authors [9,10,11]. Several investigations have concluded that the number of transducers used influences the reliability of detection [8,12].

The primary advantages of guided waves compared to bulk waves for multilayered structure inspection are that these waves can propagate much further distances with less attenuation, and long-range inspection is possible [13]. Hence, guided wave techniques could be applied for the inspections of multilayered CFRP components.

However, guided wave signals are complex to interpret due to multimode propagation, dispersive nature, and complex geometries, increasing the complexity even more [14,15,16,17,18]. An accurate interpretation of the guided wave signal is an important factor for the assessment of structural damage. Signal processing techniques such as Hilbert transform (HT) and continuous wavelet transform (CWT) have gained attention because of the powerful feature extraction of the guided wave signal. The location of the damage can be estimated by calculating the ToF between the excited and the reflected signal. The propagation time of guided waves, phase shift, and time delay can be measured using HT [19,20,21,22,23,24,25].

State-of-the-art reviews in the field of structural damage detection and localization using wavelet-based studies were conducted by several authors [26,27,28,29,30]. The detection and localization of wave-based impact damage in composite plates using CWT by analyzing the displacement of the mode shape is presented by Janeliukstis [14], and the wavelet coefficients were investigated by Fan [15]. The influence of wavelet parameters to identify damages in various composites and current trends in wavelet-based analysis on guided wave signals has been concluded by several authors [16,17,18,19,20]. The CWT obtains a linear time–frequency representation by preselecting the mother wavelet. This method can be used to analyze the magnitude and frequency components of guided wave modes. The signal-to-noise ratio (SNR) can be improved using analytical wavelets.

On the basis of dispersion characteristics, the preferred mode can be selected to reduce the complexity. The development of the SHM technique based on guided waves for damage detection requires careful numerical modeling, because finite element analysis (FEA) of guided wave propagation and mode interaction with damage can provide useful information for further analysis.

Currently, multi-sensor arrays are commonly used in SHM to improve the accuracy of damage localization. However, the weight issue of the SHM system should not be forgotten: the additional weight due to the SHM system should be as low as possible, which means that the number of sensors used should be as low as possible. Additionally, reducing the number of sensors is beneficial in terms of the amount of data to be processed. On the other hand, the accuracy of the localization of damage should not suffer when using fewer sensors.

The objective of this research is to investigate a technique in which using only two receivers, it would be possible to determine if the delamination is located in between the transmitter and the receiver or in between the receiver and the edge of the structure. In addition, the accuracy of two different signal postprocessing techniques in improving damage localization is evaluated.

## 2. Materials and Methods

### 2.1. Modeling Setup

The CFRP plate (USN150B) of 200 mm in length and 3.5 mm in thickness was selected for analysis. The CFRP panel was constructed from 18 plies, and the laminate orientation was (–45°, 0°, +45°, 0°, 90°, 0°, –45°, 0°, and +45°)^2^. The normal force was applied on an excitation zone of 8.4 mm to excite $A_{0}$ mode by setting the transmitter–receiver configuration. The specific length of the excitation zone obtained a maximum amplitude of $A_{0}$ mode. In this study, delamination-type damages were analyzed, and the length of the delamination was 20 mm. The excitation zone was at the leading edge of the plate, while three receivers were positioned at 70, 80, and 90 mm from the leading edge (*X_r_*). Two cases of delamination positions were considered: the delamination position (*X_d_*) at 40 mm, i.e., between the excitation zone and the receivers, and at 120 mm, i.e., further than the receivers. Cases studied, positions, and distances are presented in Table 1.

**Figure 1:**
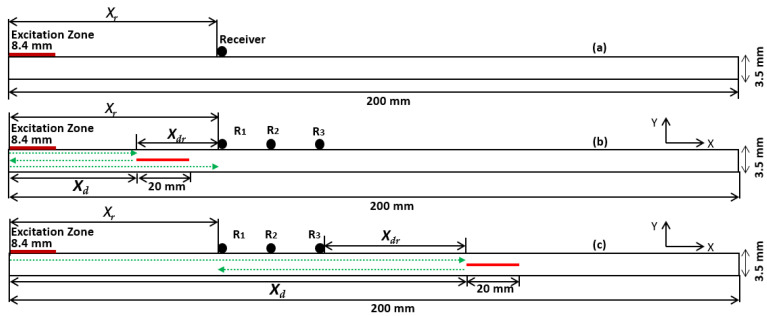
Guided wave inspection setup on a carbon fiber-reinforced polymer (CFRP) plate: defect-free plate (**a**), delamination at 40 mm (**b**), and delamination at 120 mm (**c**).

The defect-free CFRP plate is presented in Figure 1a. In case 1, the delamination is positioned in between the excitation zone and the receiver, as shown in Figure 1b. In case 2, the delamination is positioned after the receiver, as shown in Figure 1c.

In another investigation, the delamination position in the through-thickness direction was changed. The delamination position (*X_d_*) was fixed at 100 mm, and the delamination location in the through-thickness direction (*X_h_*) was modified: 1.1, 1.7, and 2.3 mm. The cases investigated are presented in Table 2.

The mechanical properties of the CFRP (USN150B) used in the numerical study are presented in Table 3. Here, *E* is the Young’s modulus, *G* is the shear modulus, ν is the Poisson’s ratio, $\rho_{c}$ is the density of CFRP, and $t_{ply}$ is the ply thickness. In this 2D model, the directions *X* and *Y* are defined as the *X*-axis along the fibers and the *Y*-axis along the thickness of the plate [21].

### 2.2. Guided Wave Dispersion Curves

It is essential to obtain dispersion curves to select suitable modes and the central frequency for the inspection [23,24,25,26]. The semi-analytical finite element (SAFE) method is one of the most common approaches to calculate eigenvalues of guided wave modes in arbitrary cross-sectional waveguides [27]. In this method, the one-dimensional cross-section of the plate is discretized, and an analytical solution is applied along the wave propagation direction. The governing equation for wave propagation in an elastic medium can be expressed by the following equation:

(1) $$\left(\lambda +\mu \right)\;\nabla \;\left(\nabla \cdot u^{\prime }\right)+\mu \nabla^{2}u^{\prime }=\rho \frac{\partial^{2}u^{\prime }}{\partial {t^{2\;}}^{\prime }}\;,$$

where λ and *µ* are the Lamé constants, ρ is the density, and *t* is the time. The $u_{x}^{\prime },\;u_{y}^{\prime },\;\mathrm{and}\;u_{z}^{\prime }\;$ are in-plane, shear horizontal, and out-of-plane displacement vectors.

In this analysis, the length and width of the plate were assumed to be infinitely long to consider the guided wave mode as symmetrically distributed in the through-thickness direction. Eighteen elements across the plate were assigned, and the thickness of one element replicates one ply of the multilayered plate. The thickness of each ply was 0.194 mm and the total thickness of the 18 plies of the CFRP plate was 3.5 mm.

**Figure 2:**
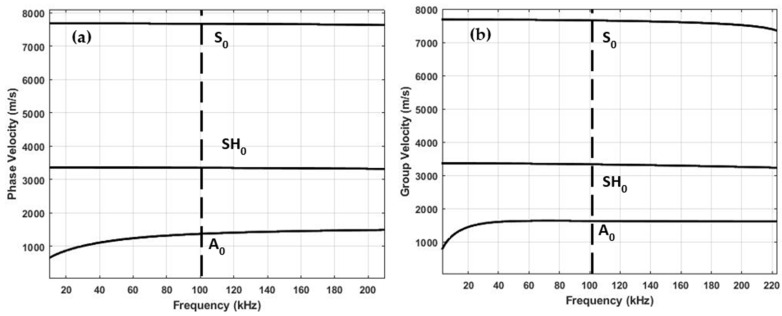
Guided wave dispersion curves in CFRP panel (USN150B): (**a**) phase velocity versus frequency and (**b**) group velocity versus frequency.

The SAFE method was derived to obtain the dispersion curves of guided wave phase velocity, $c_{ph}\left(f\right)$, and group velocity, $c_{g}\left(f\right)$, dispersion curves. The phase velocity versus frequency and group velocity versus frequency dispersion curves are presented in Figure 2a,b. From the dispersion curves, it follows that at a 100 kHz frequency, the phase and group velocities of $A_{0}$ mode were 1369 and 1625 m/s, respectively. The selected frequency range does not obtain higher-order modes and enables only fundamental modes. The maximum amplitude of each mode varies depending on the frequency. In this analysis, the guided wave mode at higher frequency had a smaller wavelength, but in return, attenuation increased, and superposition of different modes led to complex signal interpretation due to dispersion.

### 2.3. Guided Wave Modal Analysis

The purpose of the SAFE method is not only to calculate dispersion curves, but in addition, to analyze displacement distribution in the through-thickness of the plate and to select a particular mode, which would be sensitive to delamination-type defects. In guided waves, out-of-plane displacements are dominant in asymmetrical modes, and in-plane displacements are dominant in symmetrical modes.

**Figure 3:**
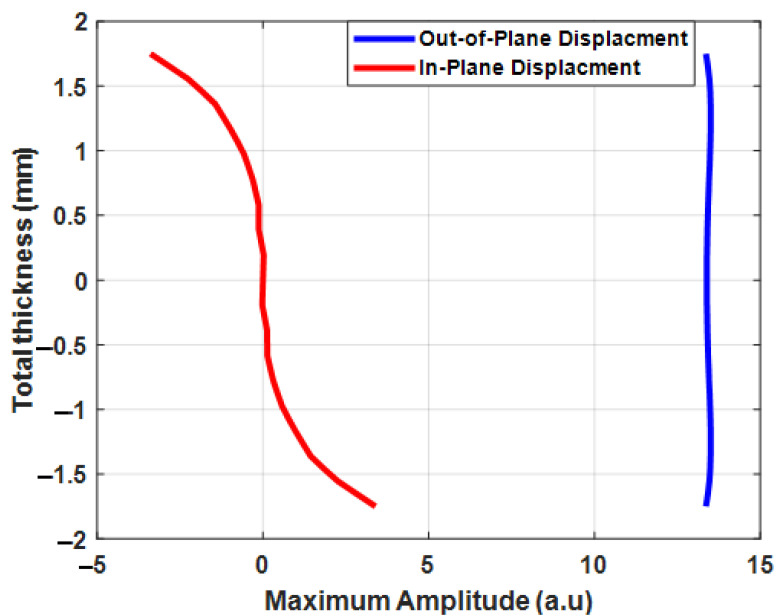
Through-thickness displacements and the maximum amplitude of the $A_{0}$ mode in the 3.5 mm-thick CFRP plate.

The in-plane and out-of-plane displacements of $A_{0}$ mode in a 3.5 mm-thick CFRP plate are presented in Figure 3. The maximum amplitude of in-plane displacements of $A_{0}$ mode at 100 kHz was relatively small compared to out-of-plane displacements of $A_{0}$ mode. Therefore, the maximum amplitude of out-of-plane displacements of the $A_{0}$ mode was dominant and relatively higher compared to in-plane displacements.

**Figure 4:**
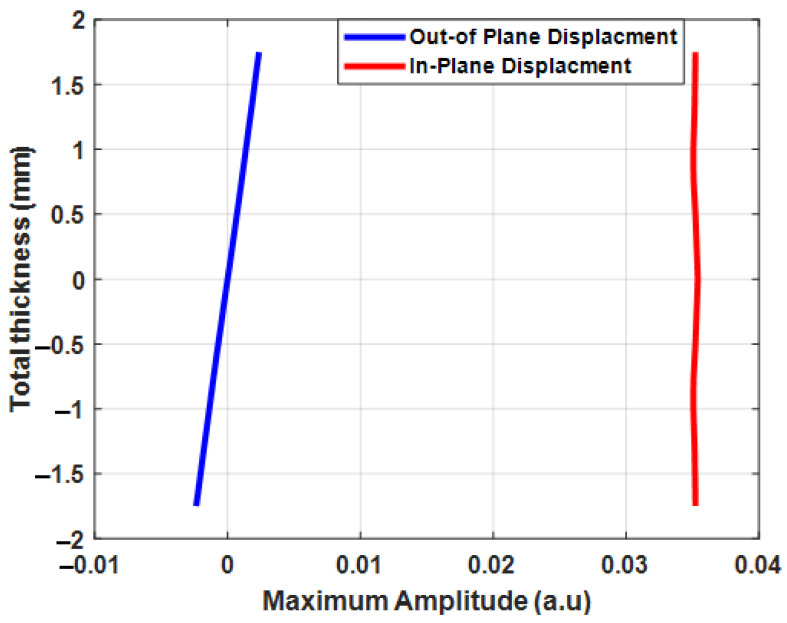
Through-thickness displacements and the maximum amplitude of the $S_{0}$ mode in the 3.5 mm-thick CFRP plate.

The in-plane and out-of-plane displacements of the $S_{0}$ mode in a 3.5 mm-thick CFRP plate are presented in Figure 4. In this analysis, the maximum amplitude of in-plane and out-of-plane displacements of the $S_{0}$ mode at 100 kHz was relatively small compared to $A_{0}\;$ mode.

The $A_{0}$ mode was selected for the inspection of the component with delamination-type defects, as the maximum amplitude of particle displacements of $A_{0}$ mode was significantly higher compared to the $S_{0}$ mode. In addition, the asymmetric mode was sensitive due to its displacement distribution in the thickness of the plate and $A_{0}$ mode velocity changes over delamination. The out-of-plane displacements of the $A_{0}$ mode were uniformly distributed throughout the thickness of the plate. The FEA of the guided wave propagation in the CFRP plate was performed to analyze guided wave interactions with delamination at different positions and depths, as presented in the following sections.

### 2.4. Numerical Simulation of Guided Waves

Experimental inspection of every guided wave propagation scenario for different damages, loading, and environmental conditions requires many mock-up trials. Therefore, physics-based numerical modeling is an efficient way to understand the mechanism of guided wave propagation and interactions with delamination in composite structures.

The guided wave interaction with delamination was analyzed with the transmitter–receiver configuration, as shown in Figure 1. A numerical simulation was performed to analyze the sensitive mode to estimate the position of the delamination in the longitudinal and through-thickness directions. The simulation was performed in the 2D approach, where the plate is infinite along the *z*-axis. The multilayered CFRP panels were anisotropic and the GW propagation velocity was angle-dependent. The 3D modeling could give propagation in all directions, which would be closer to a real investigation. However, 3D modeling of ultrasonic wave propagation, taking into account 20 elements per wavelength criterion, requires huge computational and time resources. In this research, numerical modeling was performed to investigate the interaction of guided waves with delamination-type defects at different depths. Therefore, modeling of guided wave propagation using the 2D plane strain approach was most suitable for the given task. The simulation was carried out with 2100 time-steps and the duration of each step was 0.1 µs. The total modeled duration of the $A_{0}$ mode propagation was 210 µs.

In FEA, mesh shape and size are important factors. The CPE4R-type elements were selected for the analysis in ABAQUS explicit. These types of elements are plain-strain quadrilateral and allow less integration time, and better hourglass control. In FEA, hourglass control attempts to minimize severe mesh distortion and provides better mesh control. On the other hand, a smaller mesh size improves the accuracy of the analysis but increases the computation time. In guided wave simulation, the mesh size is significantly dependent on the number of elements per wavelength (N/λ). To maintain a good balance between accuracy and computation time, 20 elements per wavelength (λ) were selected for the mesh calculation. Therefore, the mesh size was 0.6 mm.

The fundamental asymmetrical $\left(A_{0}\right)$ mode was excited by applying normal force on the excitation zone. The input signal was a five-cycle sine wave with a Gaussian envelope at a driving frequency of 100 kHz.

**Figure 5:**
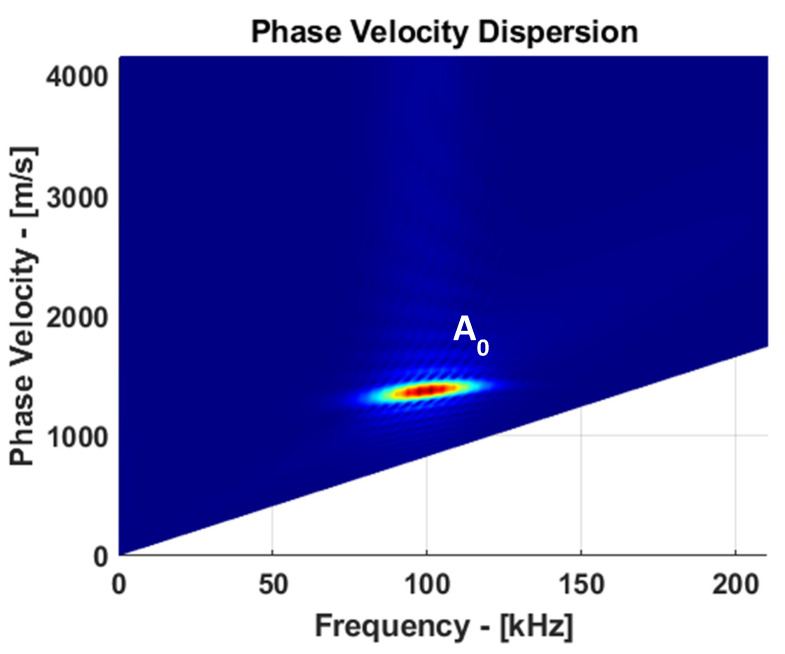
Guided wave $A_{0}$ mode phase velocity dispersion at 100 kHz.

Guided wave analysis at 100 kHz is an appropriate way to achieve a good balance between dispersion, mode complexity, and attenuation. In order to properly identify $A_{0}$ mode, the signals on the surface of the plate were recorded and corresponding B-scan data were obtained. Using 2D Fourier transform, B-scan data were converted into phase velocity versus frequency domain, as presented in Figure 5.

The dispersion characteristics of the guided waves propagating in the CFRP plate from B-Scan data were obtained using the two-dimensional Fourier transform. The propagated guided wave along the plate is expressed as a function of the coordinates and time, *u*(*x*,*t*), and can be transformed into the wavenumber, *k,* and the frequency space using 2D FFT [28]:

(2) $$H\left(k,f\right)=\int_{-\infty }^{+\infty }\int_{-\infty }^{+\infty }u\left(x,t\right)e^{-j\left(kx+\omega t\right)}dxdt$$

where *x* is the coordinate, *t* is the time, *ω* is the angular frequency, and *k* is the wavenumber.

## 3. Results

### 3.1. Guided Wave Interaction with Delamination

**Figure 6:**
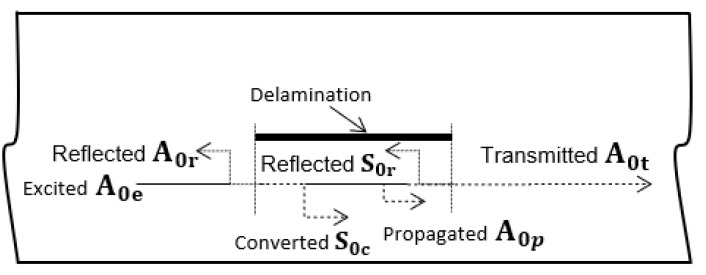
$A_{0}$ mode reflection and conversion at the leading and trailing edges of the delamination.

When the guided wave travels along the sample and encounters a delamination, part of excited $A_{0e}$ mode is reflected at the leading edge of the delamination and part is converted to $S_{0c}$ mode, as shown in Figure 6. A vertical, out-of-plane displacement component of the propagated wave was recorded in the study, as it is the dominant component of the A_0_ mode. Meanwhile, the vertical displacement component of the S_0_ mode is practically close to zero (see Figure 4). While traveling over the delamination, the converted $S_{0c}$ mode was converted back to $A_{0t}$ mode at the trailing edge of the delamination. Finally, strong reflected ($A_{0r}$) and transmitted ($A_{0t}$) modes were obtained after interacting with the delamination. Therefore, only reflected (A_0r_), and transmitted ($A_{0t}$) modes can be observed in the B-scan images.

**Figure 7:**
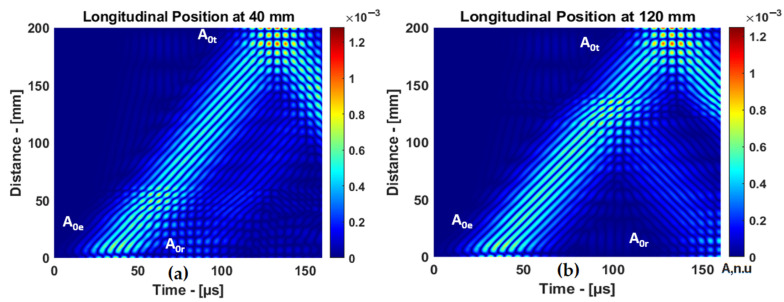
The B-scans of $A_{0}$ mode reflected from delamination at different positions: (**a**) delamination at 40 mm and (**b**) delamination at 120 mm.

The B-scans of the response of the guided wave $A_{0}$ mode to delamination at different positions (40 and 120 mm) in the longitudinal direction are presented in Figure 7. The propagation time of $A_{0}$ mode varies when the delamination position changes.

### 3.2. Localization of Delamination Location Using Hilbert Transform (HT)

The guided wave interaction with delamination at 40 and 120 mm was analyzed. To analyze the delamination position (*X_d_*), the signals were acquired at three positions of the receivers, as shown in Figure 1. In the case when delamination was in between the excitation zone and the receiver, i.e., *X_d_* was 40 mm, the guided wave signals were received (*X_r_*) at 70, 80, and 90 mm with respect to the leading edge of the CFRP plate. The delamination location can be estimated by analyzing the reflected wave from the delamination.

**Figure 8:**
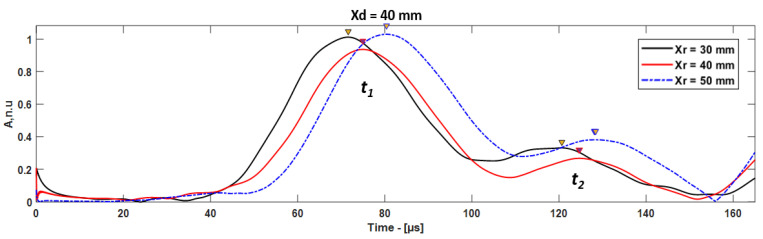
The comparison of time of flight (ToF) of $A_{0}$ mode in case of delamination at 40 mm, signals received by different receivers.

In this case, the $A_{0}$ mode propagates to the receiver, which is considered as direct $A_{0}$ mode (*t*_1_), and the reflection from the delamination must be considered as *t*_2_. The delamination position can be estimated considering the time of flight (ToF) between *t*_1_ and *t*_2_. The Hilbert envelope of the guided wave signals received at different receiver positions are presented in Figure 8. The envelope of the guided wave signal obtained using Hilbert transform, *H*(*t*), can be expressed using the following equation [29,30,31,32,33]:

(3) H(t)=|Hilbert [x(t)]|

The distance between the receiver and delamination can be estimated by calculating ToF between $t_{1}$ and $t_{2}$ [34]:

(4) $$Xe=\frac{\Delta t\cdot c_{g}}{2}\;,$$

$\Delta t=t_{2}-t_{1}$ is the time difference between direct and reflected $A_{0}$ mode, and $c_{g}$ is the group velocity.

The location of delamination was estimated by measuring the time difference between direct and reflected $A_{0}$ mode from delamination. If the delamination is located between the excitation zone and the receiver, the excited $A_{0}$ mode is reflected from the delamination leading edge, then from the leading edge of the sample, and only then does it propagate to the receiver (Figure 1b). Therefore, guided wave mode approaches delamination multiple times until it reaches the receiver. The estimation of the delamination location is presented in Table 4. In this case, the estimated distance is the delamination position from the leading edge. The relative error between the actual and the measured value can be calculated by Equation (5):

(5) $$\varepsilon_{R}=\frac{D_{a}-D_{m}}{D_{m}}\cdot 100\%\;,$$

where $\varepsilon_{R}$ is the relative error, and *D_a_* and *D_m_* are the actual and measured values, respectively.

**Figure 9:**
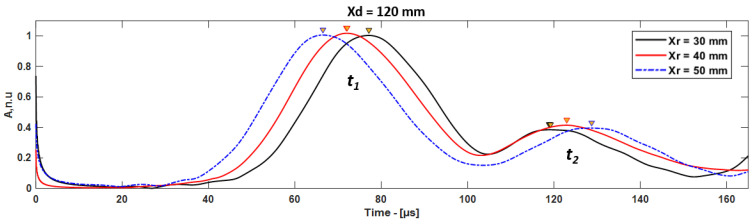
The comparison of ToF of $A_{0}$ mode in case of delamination at 120 mm, signals received from different receivers.

In the case of *X_d_* = 120 mm, the guided wave signals were received at 70, 80, and 90 mm with respect to the leading edge of the CFRP plate. The distance between delamination and the receiver (*X_dr_*) was 50, 40, and 30 mm. In this approach, if the delamination is located after the receiver position, the excited $A_{0}$ mode is received at the receiver as the first wave packet (*t*_1_) and the first reflection from the leading edge of the delamination is received as the second wave packet (*t*_2_) (Figure 1c), as shown in Figure 9.

In this case, the estimated distance is the distance between delamination and the receiver. The delamination position (*X_d_*) can be estimated by adding the distance between the edge of the sample and the receiver and the estimated distance between the receiver and delamination (*X_e_*). The localization of delamination and the relative error of this approach are presented in Table 5.

### 3.3. Localization of Delamination Using Continuous Wavelet Transform (CWT)

The wavelet analysis is one of the well-known signal processing approaches for nonstationary signals. In the CWT, a wavelet, ψ, is the analyzing function. This method compares the signal with the shift and dilation of a wavelet [35,36,37,38,39,40]. Here, dilation refers to stretched or compressed versions of the wavelet. It provides a linear time–frequency representation by pre-selecting the mother wavelet (Morse), ψ. The Morse wavelet has the advantage of determining both time and frequency components at once due to the better symmetry and time–bandwidth product of the mother wavelet. The CWT of the analytic signal to represent in the time–frequency domain can be defined by the following equation [10,41,42]:

(6) $$U\left(t,f\right)=\mathrm{CWT}\left(u\left(t,x\right)\left(f_{s}\right)\right),$$

where CWT denotes continuous wavelet transform, *t* is the time, *f* is the frequency, and $f_{s}$ is the sampling frequency.

**Figure 10:**
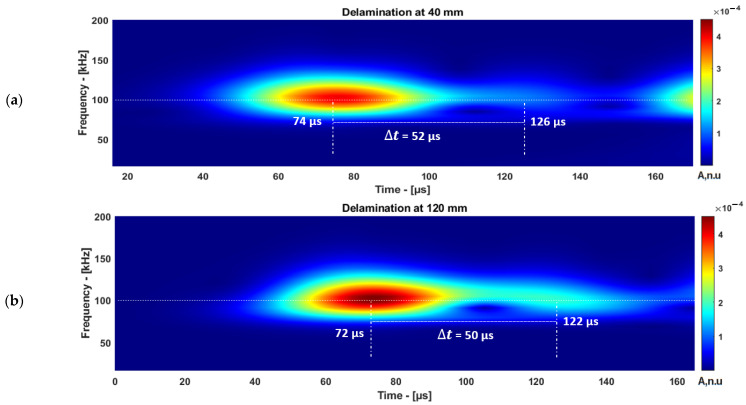
The ToF estimation of reflected $A_{0}$ mode received at 80 mm: delamination at 40 mm (**a**) and 120 mm (**b**).

The inspection setup to estimate the delamination location is presented in Figure 1b,c. The ToF of reflected $A_{0}$ mode from different delamination locations in the time–frequency domain is presented in Figure 10a,b.

The guided wave mode response obtained from the delamination position (*X_d_*) at 40 mm is post-processed and the estimated location using CWT is presented in Table 6.

The guided wave mode response obtained from the delamination position (*X_d_*) at 120 mm and the signals received at different receiver positions to estimate the delamination location using CWT are presented in Table 7.

The location of the delamination can be estimated using Equation (3). The maximum amplitude of the wave packet was used for the analysis. Therefore, the group velocity of $A_{0}$ mode was used to estimate the location of the delamination. The accuracy of the measured delamination location improved using CWT compared with HT, the comparison is presented in Table 8.

The defect location measurement using CWT provided more accurate results compared with HT. Furthermore, the time–frequency distribution of the guided wave signal along with the amplitude were obtained simultaneously. The proposed method has the potential to measure the ToF of a signal by considering the excitation frequency as a reference even in the presence of other frequency content in the signal.

## 4. Characterization of Delamination at Different Depths

In this section, the delamination position in the through-thickness direction was examined. The delamination position in the longitudinal direction was 100 mm, and the positions at different depths of 1.1, 1.7, and 2.3 mm were analyzed in the CFRP plate.

**Figure 11:**
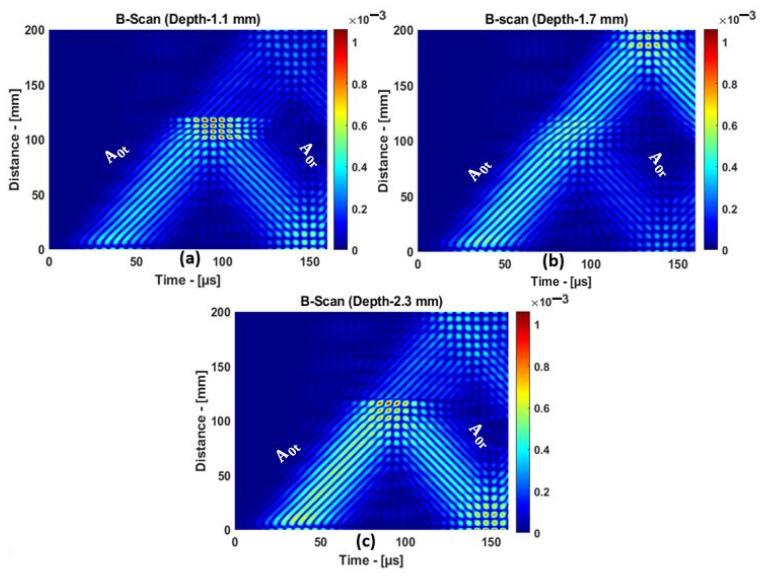
The $A_{0}$ mode reflection and transmission: delamination at (**a**) 1.1, (**b**) 1.7, and (**c**) 2.3 mm.

For 1.1 and 2.3 mm, the delamination was close to the top and bottom surfaces of the plate. For 1.7 mm, the delamination was right in the center of the plate. The B-scan representation of the $A_{0}$ mode response to delamination at different depths is presented in Figure 11a–c. From the B-scan results, strong reflections from the delamination positions at 1.1 and 2.3 mm can be observed. A weak reflection was observed when the delamination was positioned at 1.7 mm, i.e., in the middle of the sample.

### 4.1. Mode Separation

**Figure 12:**
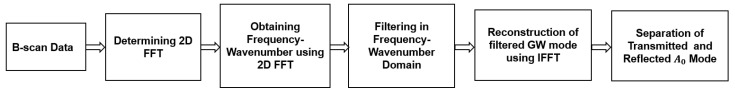
The implementation of the signal processing method for mode separation.

The amplitude of the transmitted and reflected guided wave mode varies depending on the position and size of the defect. Therefore, it is necessary to examine transmitted and reflected modes separately. In order to separate superimposed modes, the signal processing method was applied on the received guided wave signal. The flow chart of the signal processing method to separate reflected and transmitted modes is presented in Figure 12. In this analysis, the inverse fast Fourier transform (IFFT) method was used to characterize mode conversion, reflection, and transmission at the leading and trailing edges of delamination.

Initially, a two-dimensional fast Fourier transform (2D FFT) was applied to the B-scan data to obtain the wavenumber of the reflected and transmitted modes. The wavenumber of the signal using 2D fast Fourier transform can be expressed by the following equation [28]:

(7) U(f,k)=FT(u(t,x)),

where *t* is the guided wave propagation time and *x* is the propagation distance.

**Figure 13:**
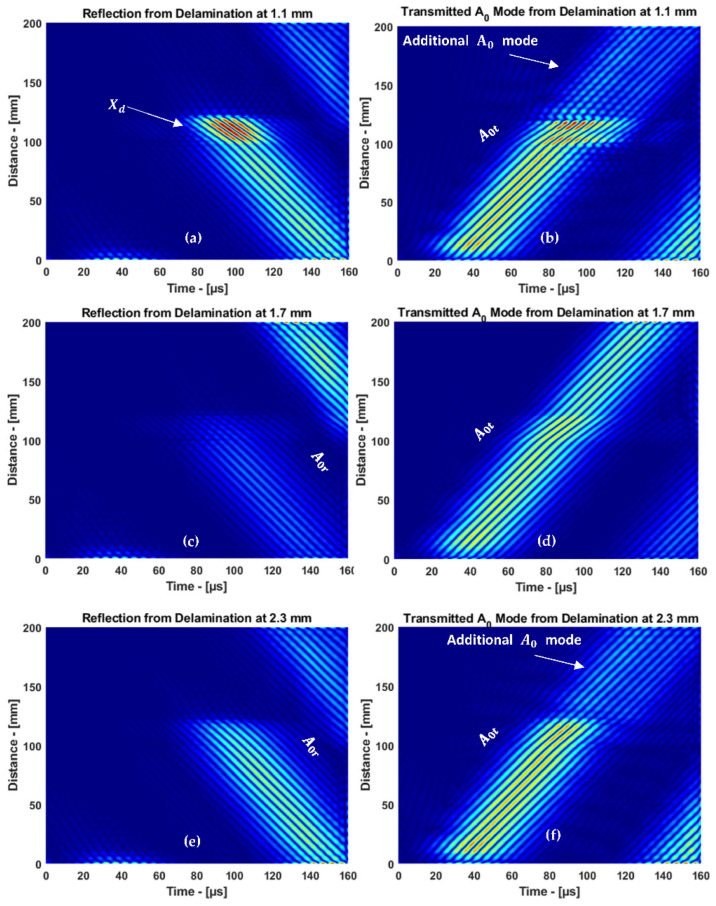
The reflected and transmitted $A_{0}$ mode through delamination: (**a**,**b**) 1.1 mm, (**c**,**d**) 1.7 mm, and (**e**,**f**) 2.3 mm.

The bandpass filter with a cosine-tapered window function was applied to the frequency–wavenumber domain to separate the modes. The wavenumber of reflected $A_{0}$ mode was isolated and the filtered mode was reconstructed in the B-scan representation using IFFT. The same procedure was followed to reconstruct the transmitted mode as well. The reflected and transmitted modes are separated and reconstructed using IFFT [28]:

(8) $$u_{F}\left(t,x\right)={\mathrm{FT}}^{-1}\left[U\left(f,k\right)\cdot B\left(f,k\right)\right],$$

where *B*(*f, k*) is the 2D bandpass filter of the selected mode. The results obtained using IFFT to separate the guided wave modes are presented in Figure 13a–e.

In the cases of delamination at 1.1 and 2.3 mm, the $A_{0}$ mode interacted with delamination and the top/bottom surface of the plate simultaneously. Therefore, an additional $A_{0}$ mode appeared in the transmitted mode, as shown in Figure 13b,f. In the case of delamination at 1.7 mm, there was a normal interaction with the top/bottom surface of the plate. Therefore, only the transmitted mode was observed. Thus, the signal processing method has shown a significant improvement to differentiate the additional $A_{0}$ mode obtained from the 1.1 and 2.3 mm delamination in the 3.5 mm CFRP plate.

### 4.2. Distinguishing Near-Surface Delamination

Guided waves are frequency-dependent, and the amplitude of each mode varies over different frequency ranges [43]. In this analysis, the reflected and transmitted $A_{0}$ mode response was presented in the frequency spectrum to estimate the amplitude. The frequency spectrum of reflected $A_{0}$ mode interacting with delamination at different depths was compared with the defect-free signal.

The amplitude of $A_{0}$ mode versus frequency can be determined from the filtered two-dimensional spectrum presented in the following equation [28]:

(9) $$U_{F}^{\prime }\left(f\right)=\underset{k}{\max}\left[U\left(f,k\right)\cdot B\left(f,k\right)\right],$$

**Figure 14:**
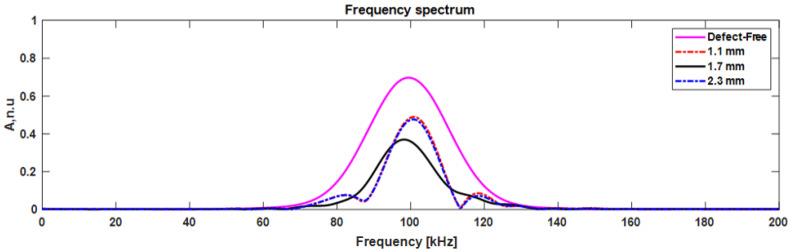
The frequency spectrum of reflected $A_{0}$ mode.

The frequency spectrum of reflected $A_{0}$ mode compared with the defect-free signal is presented in Figure 14. The reflected signal amplitude was reduced by 31% and 32% compared to the defect-free signal when the delamination was present near the top and bottom surfaces (at 1.1 mm depth), respectively. It is an indication of strong reflection, and is due to the delamination edge and surface of the CFRP plate. It is important to note that the delamination position at 1.1 and 2.3 mm has the same distance from the top/bottom of the plate. Therefore, the amplitude of the reflected $A_{0}$ mode is similar. In the case where delamination presented at the center (1.7 mm) of the plate, the signal amplitude was reduced by 48%. When $A_{0}$ mode propagated over the delamination without the interference of the boundaries, a weak reflection occurred.

**Figure 15:**
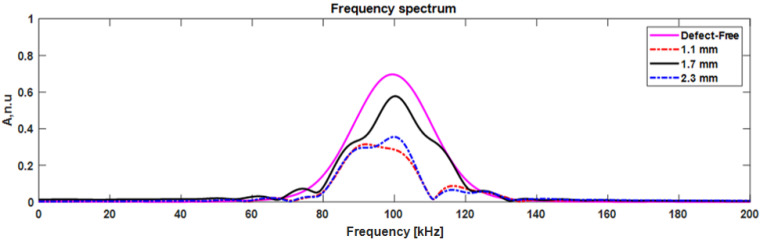
The frequency spectrum of transmitted $A_{0}$ mode.

Considering the transmitted mode, the signal amplitude was reduced by 60% and 50% when the delamination was present at the top and bottom surfaces (1.1 mm depth), respectively. As mentioned above, the delamination at 1.1 and 2.3 mm was at the same distance from the top/bottom of the plate. Hence, the transmitted $A_{0}$ mode amplitude was similar for 1.1 and 2.3 mm, as shown in Figure 15. In the case where the delamination presented in the center (1.7 mm) of the plate, the signal amplitude was reduced by 18.5%. $A_{0}$ mode propagated over the delamination with a normal interaction with the top/bottom surface of the plate.

## 5. Discussion

The guided wave dispersion curves and modal analysis were performed using the one-dimensional SAFE method. In this analysis, the guided wave was bounded by two surfaces and propagated in a single direction. However, guided wave modes have displacements in three directions and are dominant in a single direction. Therefore, from the analysis, $A_{0}$ mode had a dominant displacement in the *y*-direction and had a higher amplitude compared to $S_{0}$ mode.

The plain-strain numerical simulation was performed on a multilayered CFRP panel to analyze the guided wave mode response with delamination. In this analysis, two different inspection setups were used to locate the delamination. In one case, the delamination was located between the excitation zone and the receiver. In the other case, the delamination was located after the receiver. In the conventional way, the defect location can be estimated by measuring the ToF between the converted and transmitted modes. In this analysis, the converted $S_{0}$ mode had very low amplitude. Therefore, it was not possible to locate the delamination by evaluating converted and transmitted modes.

In guided wave inspection, the reflected mode could be useful for delamination localization when the converted mode does not provide any information due to the low amplitude. The location of the delamination can be estimated using ToF between direct $A_{0}$ mode obtained from the excitation zone and reflected $A_{0}$ mode from delamination.

In this approach, the HT and CWT were used to calculate the ToF between the direct (*t*_1_) and the reflected (*t*_2_) $A_{0}$ mode. The HT obtains the envelope of the signal, and the ToF of the signal can be estimated by measuring the peak-to-peak amplitude of the *t*_1_ and *t*_2_. The CWT obtains the time–frequency and maximum amplitude of the signal simultaneously. The ToF between the maximum amplitudes of the two wave packets allows to estimate the defect location. In addition, the CWT has the potential to achieve accurate local transients in the signal.

ToF was similar in cases when the delamination was positioned between the excitation zone and the receiver, or after the receiver. Using three receivers at different positions enabled to determine the delamination location. In the case when the delamination was in between the excitation zone and the receivers, the estimated distances for all the receiver positions were similar and showed the delamination position from the leading edge. Meanwhile, in the case when the delamination was further than the receiver, the estimated distances for the three receiver positions were different and showed the delamination position from the respective receivers.

When analyzing near-surface delamination at 1.1 and 2.3 mm in the through-thickness direction, strong reflections were obtained due to the delamination edge and the top/bottom surface of the CFRP plate. In the case of delamination at a depth of 1.7 mm, weak reflection was obtained.

When considering the transmitted mode, a weak transmitted mode was obtained due to the delamination edge and the top/bottom surface of the CFRP plate. The presented method can be used to determine the characterization of near-surface delamination, but it has limitations when it comes to measuring the exact delamination depth.

## 6. Conclusions

The $A_{0}$ mode was selected for the delamination inspection because the dominant and maximum amplitude of particle displacements of $A_{0}$ mode is significantly higher compared to $S_{0}$ mode. In addition, the asymmetrical mode is sensitive to delamination-type damages due to its displacement distribution through the thickness of the plate and guided wave mode velocity shift as it approaches delamination.

In this investigation, the inspection setup was proposed to detect and localize the delamination. This method offers to locate delamination when the converted modes do not have a significant amplitude. The reflected mode provides useful information to precisely locate the delamination. The localization of the delamination was estimated using six different scenarios to assess the accuracy of the proposed inspection setup. All inspection scenarios have shown consistent results and obtained an accurate estimate of the location of the delamination, with a relative error of less than 7.5%. Using three receivers at different positions allowed to determine where delamination was located, either in between the excitation zone and the receivers, or further than the receivers.

In this article, signal processing methods to improve damage detection and localization using guided waves have been proposed. The localization of delamination using HT and CWT was compared, where the relative error using HT and CWT was 2.5% and 1.2%, respectively. CWT obtained more accurate results to estimate the location of the defect compared to HT. In addition, CWT provided better linear time–frequency representation, higher resolution of time–frequency components, a finer spectrum, and it is superior for localizing the transients of the guided wave signal.

While characterizing near-surface delamination at 1.1 and 2.3 mm in the through-thickness direction, a strong reflection was obtained from the delamination edge and the top/bottom surface of the CFRP plate. Such a mode response occurred as a result of the variation in propagation of mode speed along the upper and lower surfaces of the delamination. In the case of 1.7 mm, the distance between the upper and lower surfaces of the plate was identical. Therefore, the mode propagation speed was identical along the upper and lower surfaces of the delamination.

Damage detection was improved using 2DFFT. When characterizing near-surface delamination at 1.1 and 2.3 mm, consecutive $A_{0}$ modes were obtained due to the boundaries and delamination. Since the consecutive $A_{0}$ modes were superimposed, it is difficult to differentiate the additional $A_{0}$ mode in the B-scan image from the reflected and transmitted modes. The additional $A_{0}$ mode was revealed after separating the reflected and transmitted modes and reconstructed using the IFFT. Thus, the complexity of signal interpretation was reduced, and damage detection was improved.

Future work will focus on the experimental verification of the proposed approach, which will be performed as the next step of this investigation.

## Figures and Tables

**Table 1:** Delamination position in the longitudinal direction.

Case	Delamination Position (*X_d_*) (mm)	Distance between Edge of the Sample and Receiver (*X_r_*) (mm)	Distance between Delamination and Receiver (*X_dr_*) (mm)
1-1	40	70	30
1-2	40	80	40
1-3	40	90	50
2-1	120	70	50
2-2	120	80	40
2-3	120	90	30

**Table 2:** Delamination position in the through-thickness direction.

Scenario	Delamination Location (*X_d_*) (mm)	Delamination Location in Through-Thickness Direction (*X_h_*) (mm)
1.	100	1.1
2.	100	1.7
3.	100	2.3

**Table 3:** The mechanical properties of the USN150B carbon fiber-reinforced polymer (CFRP) material [22].

E_1_ (GPa)	131
E_2_ = E_3_ (GPa)	8
G_12_ = G_13_ (GPa)	4.5
G_23_ (GPa)	3.5
ν_12_ = ν_13_ (-)	0.29
ν_23_ (-)	0.47
*ρ_c_* (g/cm^3^)	1544
t_ply_ (mm)	0.194

**Table 4:** The localization of delamination at 40 mm using different receivers.

Delamination Position (*X_d_*) (mm)	Distance between Edge of the Sample and Receiver (*X_r_*) (mm)	Distance between Delamination and Receiver (*X_dr_*) (mm)	ToF of First Wave Packet (*t*_1_)	ToF of Second Wave Packet (*t*_2_)	ToF (Δt) (μs)	Estimated Distance (*X_e_*) (mm)	Relative Error (%)
40	70	30	71	120	49	39	3.5
40	80	40	74	127	53	43	7.5
40	90	50	80	129	49	39	3.5

**Table 5:** The localization of delamination at 120 mm using different receivers.

Delamination Position (*X_d_*) (mm)	Distance between Edge of the Sample and Receiver (*X_r_*) (mm)	Distance between Delamination and Receiver (*X_dr_*) (mm)	ToF of First Wave Packet (*t*_1_)	ToF of Second Wave Packet (*t*_2_)	ToF (Δt) (μs)	Estimated distance (*X_e_*) (mm)	Relative Error (%)
120	70	50	66	128	62	50.3	0.6
120	80	40	72	122	50	40.6	1.5
120	90	30	77	119	42	34	13

**Table 6:** The localization of delamination at 40 mm using CWT.

Delamination Position (*X_d_*) (mm)	Distance between Edge of the Sample and Receiver (*X_r_*) (mm)	Distance between Delamination and Receiver (*X_dr_*) (mm)	ToF of First Wave Packet (*t*_1_)	ToF of Second Wave Packet (*t*_2_)	ToF (Δt) (μs)	Estimated Distance Delamination Position (*X_e_*) (mm)	Relative Error (%)
40	70	30	71	120	49	39	3.5
40	80	40	74	126	52	42	5
40	90	50	80	129	49	39	3.5

**Table 7:** The localization of delamination at 120 mm using CWT.

Delamination Position (*X_d_*) (mm)	Distance between Edge of the Sample and Receiver (*X_r_*) (mm)	Distance between Delamination and Receiver (*X_dr_*) (mm)	ToF of First Wave Packet (*t*_1_)	ToF of Second Wave Packet (*t*_2_)	ToF (Δt) (μs)	Estimated Distance between Receiver and Delamination (*X_e_*) (mm)	Relative Error (%)
120	70	50	66	128	62	50.3	0.6
120	80	40	72	122	50	40.6	1.5
120	90	30	78	118	40	32	6

**Table 8:** Comparison of HT and CWT for damage location measurement.

Delamination Position (*X_d_*) (mm)	Distance between Edge of the Sample and Receiver (*X_r_*) (mm)	ToF (Δt)	Localization Using HT (mm)	Relative Error Using HT (%)	ToF (Δt)	Localization Using CWT (mm)	Relative Error Using CWT (%)
40	40	53	43	7.5	52	42	5
120	40	51	41	2.5	50	40.6	1.5

## Data Availability

Not Applicable.
